# Septic Hip Revision Arthroplasty—A Perioperative and Follow-Up Risk Analysis

**DOI:** 10.3390/jcm13206202

**Published:** 2024-10-18

**Authors:** Julius Borkens, Christian Götze, Filippo Migliorini, Cueneyt Sönmez, Julian Koettnitz

**Affiliations:** 1 Faculty of Medicine, Ruhr-University Bochum, Universitätsstraße 150, 44801 Bochum, Germany; julius.borkens@ruhr-uni-bochum.de (J.B.); christian.goetze@sfh-muenster.de (C.G.); 2 Department of Orthopaedics and Trauma Surgery, University-Clinic Aachen, RWTH Aachen University Clinic, 52064 Aachen, Germany; migliorini.md@gmail.com; 3 Department of General Orthopaedics, Auguste-Viktoria-Clinic Bad Oeynhausen, University Hospital of RUB-Bochum, Am Kokturkanal, 32545 Bad Oeynhausen, Germany; cueneyt.soenmez@muehlenkreiskliniken.de

**Keywords:** age, blood transfusion, hip, pathogens, perioperative complications, gender differences, septic revision THA

## Abstract

**Background:** Septic hip revision arthroplasty is a complex procedure associated with significant perioperative risks. This study aimed to analyze perioperative and follow-up risk factors in patients undergoing septic hip revision arthroplasty. **Methods:** A retrospective analysis was conducted on 96 patients who underwent septic revision total hip arthroplasty between 2018 and 2021 at a university hospital. Demographic data, surgical details, pathogen analyses, and complication data were collected and analyzed. The first and second hospitalizations were investigated. Data analyses were conducted with SPSS Version 29.0. **Results:** The mean age of patients was 69.06 ± 11.56 years, with 59.4% being female. On average, 1.3 ± 0.8 pathogens were detected per patient. *Staphylococcus* species were the most common pathogens. Women experienced significantly more complications during the first revision hospitalization (*p* = 0.010), including more surgical (*p* = 0.022) and systemic complications (*p* = 0.001). Anemia requiring transfusion was more common in women (70.1% vs. 43.5%, *p* = 0.012). A higher BMI was associated with a higher count of pathogens (*p* = 0.019). The number of pathogens correlated with increased wound healing disorders (*p* < 0.001) and the need for further revision surgeries (*p* < 0.001). **Conclusions:** This study identifies gender as a significant risk factor for complications in septic hip revision arthroplasty. Female patients may require more intensive perioperative management to mitigate risks. The findings underscore the need for personalized approaches in managing these complex cases to improve outcomes.

## 1. Introduction

Periprosthetic joint infection (PJI) remains one of the most challenging complications following total hip arthroplasty (THA), often necessitating revision surgery [1]. Septic revision THA is a complex procedure associated with significant morbidity and mortality risks [2,3]. While a two-stage revision has traditionally been considered the gold standard for treating chronic PJI, some specialized centers have reported comparable outcomes with one-stage exchange arthroplasty in carefully selected patients [4]. The management of septic hip revisions requires careful perioperative planning and risk stratification. However, there remains a need for a comprehensive analysis of the perioperative and long-term risks specific to septic hip revisions [5,6]. Understanding the risk profile and outcomes of septic hip revisions is crucial for optimizing surgical protocols, managing patient expectations, and potentially reducing the substantial economic burden associated with these complex cases. Previous research has identified several factors that influence the outcomes of septic hip revisions, including patient comorbidities, causative organisms, the timing of the intervention, and the surgical technique. Factors such as advanced age, obesity, diabetes, and immunosuppression have been associated with an increased risk of complications and treatment failure [2,3,7,8]. The microbiology of the infection can play a crucial role as well, with certain organisms like methicillin-resistant *Staphylococcus* aureus (MRSA) and Gram-negative bacteria posing challenges for eradication and successful revision [9,10]. Many existing studies focus on either the short-term outcomes or long-term survival rates, but few provide a detailed examination of the entire patient journey from initial presentation through follow-up [11,12]. Understanding the risk profile and outcomes of septic hip revisions is crucial for several reasons. First, it can inform preoperative risk stratification and patient counseling, allowing for more accurate prognostication and management of expectations. Second, identifying modifiable risk factors may guide the development of targeted interventions to improve outcomes. This study aims to conduct a detailed perioperative and follow-up risk analysis of septic hip revision arthroplasty cases at a single academic center in Germany. By examining a wide range of factors including patient demographics, surgical approach, microbiology, complications, and functional outcomes, we hope to provide valuable insights to guide clinical decision-making and improve patient care. Our analysis will encompass both the initial revision procedure and subsequent hospitalizations, allowing for a comprehensive assessment of the challenges and outcomes associated with septic hip revisions.

## 2. Materials and Methods

### 2.1. Study Design

**Figure 1:**
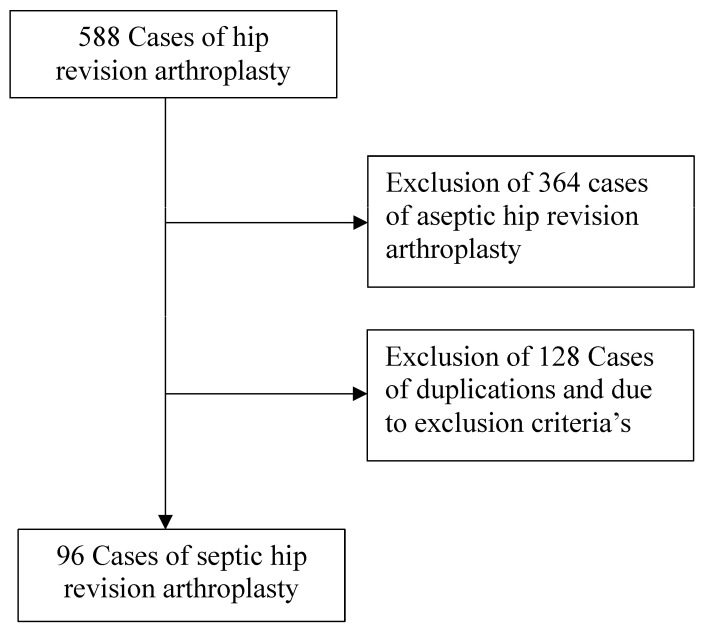
Flow chart of study design.

The present study was performed according to Strengthening the Reporting of Observational Studies in Epidemiology (STROBE) [13]. This study was conducted in the Department of Orthopedic Surgery of a university hospital in Germany. The study was conducted in accordance with the Declaration of Helsinki and approved by the local Ethics Committee of the university hospital. For more details, see Figure 1 below.

Data from patients who underwent septic revision THA from 2018 to 2021 were retrieved. The data were retrieved using Pegasos 7 (Nexus Marabu GmbH, Berlin, Germany) and collected in Microsoft Excel Version 16.89.1 (Microsoft Corporation, Redmond, WA, USA). The following data were collected at admission: age, sex, side, body mass index (BMI), length of hospital stay, length of intensive care unit stay, and American Society of Anesthesiologists physical status (ASA). The ASA classification counts from 1 to 6 (normal health, mild, severe, severe with life-threatening conditions, moribund diseases, and brain dead) [14]. The following data were collected during the hospitalization: complete knee replacement or partial component replacement (femoral or tibial replacement), preoperative and postoperative hemoglobin (Hb), the incidence of systemic and surgical complications, and the frequency of blood unit transfusions. Systemic complications included pulmonary, cardiac, urogenital, and neurologic complications. Surgical-related complications included early infections, neurologic disorders, fractures, bleeding, aseptic loosening, surgical interventions post-surgery, and post-discharge complications such as infections or instabilities.

#### Exclusion Criteria

If patient data were not accessible, the patient was excluded from the present investigation.

Patients with aseptic revision hip arthroplasty, including aseptic loosening, periprosthetic fracture, or wear of the prosthetic components, were also excluded. In addition, patients who were admitted to a German university hospital for reimplantation of the hip prosthesis but who had previously had the infected prosthesis removed in another hospital were excluded. Furthermore, patients with low and incomplete data collection were excluded, for example, due to a premature transfer.

### 2.2. Inclusion Criteria

All patients undergoing septic revision THA were retrieved, and their eligibility was assessed. The inclusion criteria were (1) patients with primary THA and clinical symptoms of a THA infection; (2) patients aged between 40 and 100 years; (3) accessible patient data; (4) revision surgery; (5) patients with a proven positive pathogen test result; and (6) patients with all primary approaches (dorsal, lateral, anterolateral, anterior).

### 2.3. Perioperative Management

The revision arthroplasties were conducted with the Zimmer Biomet Zweymueller system (Zimmer biomet, Warsaw, IN, USA) Implantcast Mutars RS system (Implantcast, Hamburg, Germany), and Link MP Reconstruction System (Link, Hamburg, Germany). For revision THA, the Bauer approach, Moore approach, and anterolateral approach were used. Every patient received a urinary tract catheter before the revision surgery. Removal was carried out as early as possible and on an individual basis, but at least after sufficient mobilization had been achieved. All patients received general anesthesia and at least short monitoring after the operation. A stationary or ambulant rehabilitation program was organized prior to hospital release.

#### Blood Unit Supply

The indication for the blood unit transfusion was according to the restrictive Cochrane guidelines: Hb-levels over 8.0 g/dL indicated no transfusion; levels between 7 and 9 indicated concomitant clinical symptoms such as dizziness, nausea, malaise, or loss of appetite; and Hb-levels under 8 g/dL indicated transfusion [15].

### 2.4. Statistical Analyses

All statistical analyses were performed using the software IBM SPSS version 29 (IBM, Armonk, NY, USA). Metric-scaled data were analyzed by mean, standard deviation, and variance. Nominal, dichotomous data were analyzed by Fisher’s exact test. For the analysis of metric and nominally scaled variables, the T-test for independent samples, variance analyses, the Levene test, and the Welch test were used. Cohen’s d (small 0.20; medium 0.50; large 0.80) and a 95% interval were used as effect sizes. The effect size used was phi (small 0.10; medium 0.30; large 0.50). For correlation analyses of metric and ordinal scales, the bivariate Pearson and Spearman correlation analyses were used. For nominal and ordinal scales, the Mann –Whitney U test and the Kruskal–Wallis test were used. The significance level was set to two-sided with α = 0.05.

## 3. Results

### 3.1. Recruitment Process

In total, data from 96 patients with septic revision THA were retrieved from 2018 to 2021.

### 3.2. Patient Demographics

Patient demographics were sorted from hospital data of the first stay to the follow-up period. See more details in Table 1.

The indications for revision were pain (27.1%), positive pathogen detection in punction (12.5%), persistent wound secretion (21.9%), positive inflammation signs (10.4%), persistent fever (6.3%), subcutaneous abscess (2.1%), luxation (7.3%), persistent fistula (5.2%), and periprosthetic fracture (2.1%). For component changes, see Table 2.

### 3.3. Pathogen Analyses

**Figure 2:**
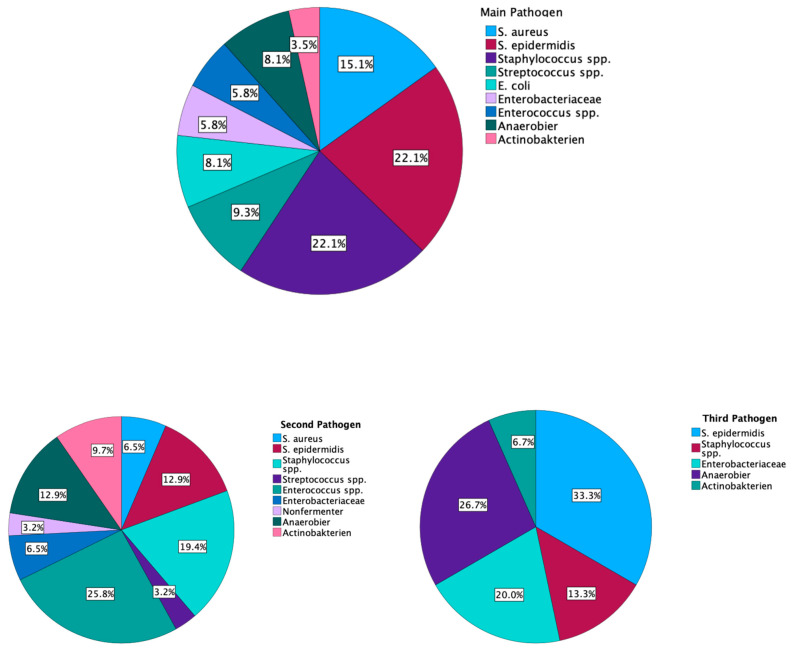
Pathogen distribution in the first hospitalization; *Staphylococcus* spp. Includes all different *staphylococcus* except *S. aureus* and *S. epidermidis*.

The analyses of pathogens identified up to three pathogens per patient. The main pathogen was found in 3.6 ± 2.4 test samples. The mean proportion of the main pathogen was 69.7 ± 30.9%. On average, 1.3 ± 0.8 pathogens were detected. No gender or age difference was found in the number of pathogens per patient (*p* = 0.517; d = 0.135/*p* = 0.308; d = 0.194), whereas a significant difference between a BMI lower than 25 and a BMI higher than 25 could be detected. A high BMI was associated with a higher count of pathogens in the infection (*p* = 0.019; d = 0.596). No difference was found between the main pathogens and the time to the first revision operation (*p* = 0.906; η^2^ = 0.042). In the second hospitalization, in 79.2% of the cases, no pathogen was found; in 2.1% of cases, the same pathogen from the first hospitalization could be found; and in 15.6% of cases, a new pathogen was detected. In 28% (*n* = 13) of patients with THA reimplantation (in the second hospitalization) (*n* = 46), a pathogen was detected. The higher the number of pathogens found in the revision surgery, the more often wound healing disorders occurred (*p* ≤ 0.001; d = 0.900) and the more often further revision surgeries were necessary (*p* ≤ 0.001; d = 0.910). The pathogen distribution is presented in Figure 2.

### 3.4. Gender Analyses

There was no difference between the sexes in the frequency of the change of mobile components (*p* = 0.417; phi = 0.158), but men were more often implanted with a spacer than women during the first revision surgery (*p* = 0.022; phi = 0.243). No significant gender difference was found in the frequency of the type of pathogens, although *Staphylococcus* species were found in women more often in fixed numbers (*p* = 0.425; phi = 0.311). No significant gender differences were detected in the pre- and postoperative walking distances during the first revision stay. Women experienced more complications including systemic and surgery-related complications during the first revision hospitalization (*p* = 0.010; phi = 0.285), but no gender differences were revealed for the follow-up complications after the second revision (*p* = 0.400; phi = 0.095). Additionally, women were significantly more likely to suffer from surgical complications (*p* = 0.022; phi = 0.251). The same observation was made regarding systemic complications (1. Hospitalization *p* = 0.001; phi = 0.347; 2. Hospitalization *p* = 0.674; phi = 0.051). Anemia was significantly higher in women than men in the first hospitalization (*p* = 0.012; phi = 0.266); in the second hospitalization, no gender differences were found (*p* = 1.0; phi = 0.022). Men more often received reimplantation in the second hospitalization (*p* = 0.36; phi = 0.228).See more details in Table 3.

### 3.5. Survival Rate

**Figure 3:**
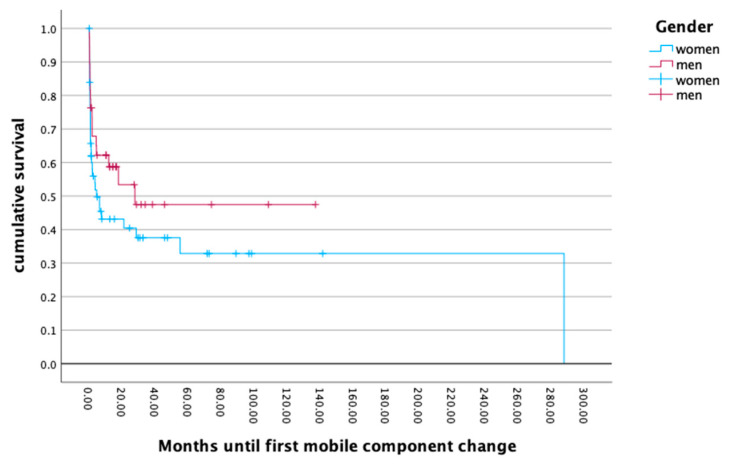
Months until first mobile component change.

**Figure 4:**
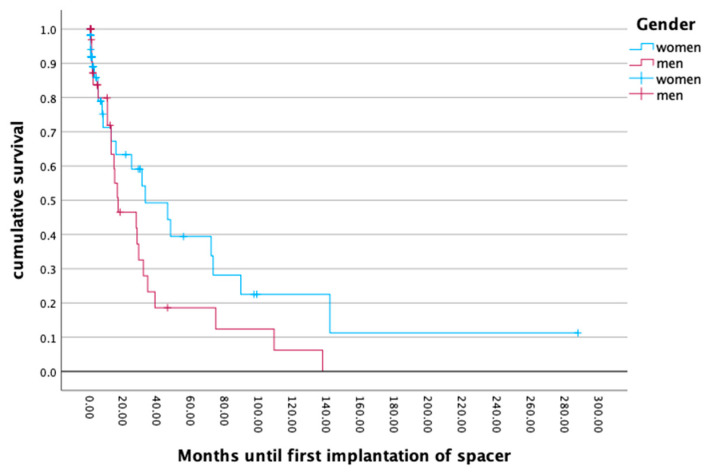
Months until first implantation of spacer.

The survival of the first implanted endoprosthesis to the first event of a mobile component change or a full component removal with implantation of a temporary spacer revealed no gender differences (*p* = 0.173; *p* = 0.120). The mean survival rate of the endoprosthesis also showed no gender differences (*p* = 0.704), with a mean for men of 19.26 months and 22.53 months for women. The cumulative survival rate revealed a periprosthetic infection rate of 50.0% at 20 months after primary implantation. An analysis of the differences in the survival rates for low and high body mass indices did not reveal any significant outcomes (*p* = 0.917). For more details, see Figure 3 and Figure 4.

## 4. Discussion

This study provides valuable insights into the perioperative risks and outcomes associated with septic hip revision arthroplasty. The findings highlight several key factors that influence complications and patient outcomes, with important implications for clinical practice.

One of the most striking findings of this study is the significant gender difference in complication rates. Women experienced more complications during the first revision hospitalization, including higher rates of surgical and systemic complications. Additionally, our study found that a higher BMI was associated with a higher count of pathogens in the infection, whereas gender did not show any differences. This aligns with previous research suggesting that obesity is a risk factor for periprosthetic joint infection and may complicate treatment [16]. Higher pathogen counts were also accompanied by worse wound healing and more frequent revision surgeries. A reason for this could be the difficult intraoperative tackling of the pathogens, especially of the small colony variants. Another factor could be antibiotic therapy, which depends on many factors, like the patient’s weight, allergies, compatibility, and the antibiotic resistogram. The treatment of different pathogens at the same time is a difficult task. Surprisingly, Petrie et al. (2023) claimed that systemic antibiotics are not required for a successful two-stage revision. Aggressive debridement and local antibiotics without prolonged systemic antibiotics were equally successful. There may be a question regarding the missing treatment of biofilm-building bacteria and possible hematogenous spreading [17] Other study groups have shown good results with systemic antibiotic therapy [18,19,20,21]. The microbiology results in our study, with *Staphylococcus* species being the most common pathogens, are consistent with the literature on periprosthetic joint infections. Gender disparity in outcomes following septic hip revision has not been widely reported in previous studies in the literature [22,23]. While some studies have found gender differences in outcomes following primary total hip arthroplasty, the specific impact on septic revisions has been less clear. Despite women being more prone to complications in the first and second hospitalization, men more often received a direct implantation of a spacer in the first hospitalization. One reason for this could be a worse infection situation or a longer wait until the decision to go to the hospital is made. Another reason could be a sex difference in the immune response. Some studies have revealed different immune responses in favor of women. This allows females to have better pathogen clearance but also leads to faster and worse inflammatory signs [24,25,26]. Offner et al. (1999) observed gender differences in the occurrence of complications after trauma surgery [27]. These results show the complexity of gender differences in clinical medicine. Orthopedic surgery should pay more attention to this factor. In addition, women have been described as an independent risk factor for periprosthetic infections in previous investigations [22,28]. On the other hand, research by Triantafyllopoulos et al. (2015) on risk factors for early PJI after total joint arthroplasty found that the male gender was actually associated with a higher risk of early infection [29]. This is contrary to findings in other studies [30,31,32,33]. Regarding urinary tract infections, women were only affected by the complication (*p* = 0.003) in the first hospitalization. The complication rate drops clearly during the second hospitalization. This could be due to a long pretreatment with antibiotics and a repaired prosthesis infection before reimplantation of the new implants. A comparison of the bacteria from the urinary tract catheter (UTC) and the pathogen of the joint infection would have been useful to find a connection. Magliano et al. (2012) described that women in the age range of 16–64 experience significantly more urinary tract infections than men. Gram-negative pathogens were found in 90% of cases [34]. The urethra of women is shorter than men, which is proposed as a reason why it is easier for ascending bacteria to reach the bladder. Another problem is the close distance of the anus to the urethra [35]. Other study groups revealed that women were more prone to urinary tract infections with UTCs [36,37]. The fact that urinary tract infections occurred in spite of antibiotic treatment and standardized hospital care allows room for many interpretations. The extended period spent in bed and the extended placement of the catheter, coupled with the female anatomy, are to be seen as the most likely causes. As a consequence, bladder catheters in women have to be removed more quickly, and remobilization should be encouraged. The survival analysis of the first implanted prostheses revealed a concerning trend, with a 50% infection rate at 20 months after primary implantation. It should be added that the patients were presented from any hospital in the region, which could have caused a bias effect. In addition, due to the hospital’s single status specialization and certification in treating prosthesis infections within a wide radius of the region, severe cases are referred directly to it. Further investigations will be carried out to find a reasonable explanation for this effect. Overall, this is substantially higher than rates reported in some previous studies [38]. For instance, other study groups found a re-revision rate of 7–30% within one year after septic revision THA in a large registry study [3,39]. The higher rate in our study may reflect potential differences in patient populations and treatment protocols. During the second hospitalization, the rate of complications decreased significantly, which demonstrates that a standardized procedure is essential for a good outcome.

While this study provides valuable insights, it has several limitations. The retrospective design and relatively small sample size (*n* = 96) limit the generalizability of the findings. Additionally, the single-center nature of the study may not capture variations in practice across different institutions. Future research should focus on prospective, multi-center studies to validate these findings and explore regional variations in outcomes. Future studies should also investigate the underlying mechanisms for the observed gender differences in complications, as well as evaluate targeted interventions to reduce complication rates, particularly in high-risk groups such as women and patients with a high BMI. There should also be long-term follow-up studies to assess the impact of perioperative factors on long-term outcomes and quality of life.

## 5. Conclusions

In conclusion, this study highlights the complex nature of septic hip revision arthroplasty and identifies several key risk factors for complications. The findings underscore the need for personalized approaches to perioperative management, particularly for female patients and those with a high BMI, especially in postoperative treatment with possible complications like urinary tract infections. By addressing these risk factors, clinicians may be able to improve outcomes and reduce the burden of complications associated with this challenging procedure.

## Figures and Tables

**Table 1:** Patient demographics. (d) = days.

Demographics	
Age	69.06 ± 11.56 years
Men	40.6% (39 of 96)
Women	59.4% (57 of 96)
BMI	30.46 ± 6.6 kg/m^2^
ASA score	2.3 ± 0.51
Pre-diseases	4.9 ± 2.6
Hospital data	
Length in days until first revision	636.8 ± 1224
Surgery time (min)	123 ± 81.65
Length of hospital stay (d)	28.3 ± 18.8
Number of surgeries in first hospital stay (mean)	2.01 ± 1.87
Number of pathogens detected	1.3 ± 0.82
Detection of the main pathogen in X samples	3.6 ± 2.42
Duration of antibiotic therapy	40.33 ± 23.09
Days of intensive care	2.84 ± 3.80
Number of blood unit transfusions	3.47 ± 5.59
Follow-up period (d)	219 ± 290
Number of follow-up stays (mean)	0.85 ± 0.89
Number of follow-up surgical interventions (mean)	1.13 ± 1.47

**Table 2:** Type of operation during first revision hospitalization and second hospitalization.

	Change of mobile components		Direct implantation of spacer		Girdlestone
1. hospitalization	51 (53.1%)		45 (46.9%)		0%
	Implantation of spacer	Reimplantation of arthroplasty	Girdlestone	Permanent fistula	Amputation
2. hospitalization	7 (7.3%)	45 (46.8%)	7 (7.3%)	2 (2.1%)	2 (2.1%)

**Table 3:** Surgery-related and systemic complications in first and second hospitalization: cardiac disorders included cardiac arrhythmias and decompensation; pulmonary disorders included pneumonia or respiratory dysfunction. * = significantly different.

	Women (*n* = 57)	Men (*n* = 39)	*p*
1. Hospitalization			
Intraoperative fracture	12.2%	2.5%	0.13
Intraoperative vascular damage	3.5%	2.5%	1
THA dislocation	10.5%	10.2%	1
Wound healing disorder	41.1%	25.8%	0.03 *
Postoperative neurologic disorders	5.2%	5.1%	1
Change of spacer (when implanted in the first surgery)	15.7%	18.7%	0.43
Complete removal of endoprosthesis	15.7%	17.9%	0.787
Permanent fistula tract	5.2%	0.0%	0.269
Anemia requiring transfusion	70.1%	43.5%	0.012 *
Blood unit transfusion (mean)	4.33	2.19	0.054
Urinary tract infection	19.2%	0.0%	0.003 *
Postoperative thrombosis	0.0%	2.5%	0.406
Renal insufficiency	0.0%	7.6%	0.064
Cardiac disorders	5.2%	2.5%	0.644
Pulmonary disorders	7.0%	5.1%	1
Postoperative delirium	3.5%	0%	0.51
2. Hospitalization	Women (*n* = 56)	Men (*n* = 38)	*p*
Intraoperative fracture	1.7%	0.0%	1
Intraoperative vascular damage	0.0%	0.0%	/
THA dislocation	12.7%	5.2%	0.301
Wound healing disorder	16.3%	18.4%	0.788
Postoperative neurologic disorders	12.7%	2.5%	0.135
Reinfection of the new implant	16.3%	18.4%	0.788
Evidence of new bacteria compared to the last hospitalization	16.3%	15.7%	1
Anemia requiring transfusion	36.3%	34.2%	1
Urinary tract infection	7.1%	0.0%	0.142
Postoperative thrombosis	3.5%	2.6%	1
Renal insufficiency	0.0%	0.0%	/
Cardiac disorders	3.5%	2.6%	1
Pulmonary disorders	3.6%	2.6%	1
Postoperative delirium	1.7%	0.0%	1
Number of follow-up stays (mean)	0.78	0.94	0.391

## Data Availability

Data generated or analyzed during this study are included in this published article. No Supplementary datasets were used for the manuscript.
